# Exploring Factors Behind Offline and Online Selfie Popularity Among Youth in India

**DOI:** 10.3389/fpsyg.2018.01403

**Published:** 2018-08-07

**Authors:** Sanchita Srivastava, Puja Upadhaya, Shruti Sharma, Kaveri Gupta

**Affiliations:** ^1^Independent Researcher, New Delhi, India; ^2^Jamia Millia Islamia Central University Delhi, New Delhi, India

**Keywords:** selfie, selfie users' perspective, online, offline, taking, posting, popularity

## Abstract

“The Selfie Culture,” practiced globally, is gaining popularity with each passing day. Owing to its ubiquitous fame across the globe, it becomes essential to inquire the grounds for such worldwide recognition. In few years, it also became the center of attraction among researchers and previous studies had recognized two important aspects of selfie: first, why is selfie posting on social media is increasing day by day and second, who choose to involve more frequently in selfie posting behavior on social media? However, these studies focused only on its online popularity on various social media platforms but did not pay much attention on its offline popularity among selfie takers. In addition to this, the multifaceted sides of selfie which may make it different from other pictures and might also play an important role in its popularity in both offline and online platforms remained unexplored. The present study addressed this gap and explored two important aspects of selfie related behavior, First, it emphasized the significance of understanding the user's conception of selfie and second, it explored the determining factors behind both offline (taking) and online (posting) modes of the practice. 60 college going students (44 females and 16 males) living in Delhi, India participated in the study. Semi-structured interviews were conducted and qualitative thematic analysis was used to cull out the themes. The results showed five factors (looks good, keeping memories, mood driven, mirroring the self and posting on social media) behind selfie offline (taking) involvement. Further, the online (posting) selfie popularity had been driven by three factors (social approval, being the best among the rest, to maintain online presence). Participants' popularity of selfie usage in both offline and online modes advocates the need to explore the offline selfie involvement of selfie takers in future research. The study also extended the existing conceptualization of selfie phenomenon which could help to unravel its wide popularity among its users.

## Introduction

“Selfie” is generally referred to as a self-generated picture taken with a Smartphone and shared on social media (Oxford Dictionaries, 2013). This self-generated “selfie” has gained immense popularity in few years. It has made a revolutionary mark among Facebook, Twitter, Instagram and Snapchat users in social media (Mike, 2014; Lim, 2016; Piwek and Joinson, 2016). As Lim (2016), (p. 1776) has also mentioned that “Today, more than a million selfies are taken each day, and more *than 17 million selfies are uploaded and shared (i.e., a selfie can be shared by the photographer and other audiences on multiple platforms, e.g., Facebook, Instagram and Twitter) on social media every week.”* Due to its marked popularity, selfie has found its significance not only in academic research, but is also used by businesses and corporate to promote them (Lim, 2016). Many Smartphone companies had begun emphasizing the best selfie features in their phone advertisements in India (such as Samsung, Oppo, Vivo, and Gionee). As recently, India has become the third country after USA and China for its largest Smartphone users (Rai, 2016). Furthermore, popular television shows have also started adding selfie activity in their shows. Moreover, Political figures and celebrities are also seen taking and sharing selfies often to register their presence on various events and occasions (Collings, 2014; Baishya, 2015; Pal, 2015). To add more on the celebrities influence on selfie popularity, Kedzior and Allen (2016) (p. 1898) mentioned about a popular celebrity, “*Kim Kardashian is probably the figure that comes most readily to mind as an influencer of the selfie practices of many, not only in terms of legitimating the style of practice itself, but often capitalizing on and reinforcing the aesthetics of male gaze.”*

In 2013, an article written by Hall (2013) for “The Telegraph” mentioned about the changing trend of photography as now young people prefer to take selfies rather than being clicked by photographer. The culture of keeping family albums is now fading among youths and mostly they like to post their selfies online and maintain online albums. The reasoning behind this changing trend could be understood by extending the definition of a photograph, that may be earlier perceived as frozen memories. As Belk (2013) advocated that photograph could be seen as a significant medium for communication and identity construction on social media. This communication could have been easy after digital social media and smartphone evolution (Ibrahim, 2015). As these digital possessions could be easily stored and accessed on computers as well as smartphones (Belk, 2014). To suffice the growing need to store numerous selfies and provide additional storage capacity to individuals, a recent application “Google photos” with high storage capacity was introduced. To a great surprise, around 24 billion selfies were uploaded in this application only in a period of 1 year (Gray, 2016). In a research article, Hess (2015) had also emphasized on people's preference to keep on taking their selfies until they got a perfect angle which ensured a best selfie. Criteria to be called good selfie and features for the best angle for selfie taking were also explored (Lin and Yeh, 2014). All these articles suggest the popularity and preferences of selfies among its users. Researchers had also attempted to examine selfie and its application from various perspectives.

The existing literature on selfie research suggested two important areas that needed investigation in order to unravel the reasons behind its huge popularity and also to address the gaps in literature:

1. Conceptualization of selfie in social media research
2. Determinants associated with selfie popularity among its users

### Conceptualization of selfie in social media research

The idea of “selfie” is not new rather has ancient roots ranging from artists painting self-portrait's [such as Van Gogh, Chimenti etc.] to scientists' innovative discoveries [such as Wheatstone, Emerson etc.] to take self-portrait by camera (Wade, 2014). However, the term “selfie” was formally incorporated as an English word in the Oxford dictionary in the year 2013. According to Oxford Dictionaries (2013) selfie is “*a photograph that one has taken of oneself, typically one taken with a smart phone or webcam and uploaded to a social media website*.” This paper argues the need to develop and understand the selfie phenomenon by extending its existing conceptualization. The question arises that why is there any need to expand the selfie phenomenon at all? Selfie research is growing proportional to its popularity among its users. Currently, around 350 millions hastags on “Instagram” related to the word “selfie” are available. This number is kept on increasing with each passing day on various social media platforms. On the other hand, if one looks into the “Scopus” search engine for selfie related papers it will show more than 1,100 research articles and book chapters within a span of 3–4years. Close to 800 articles in secondary sources and nearly 1,500 patents related to selfie. Another popular search engine existing in today's time such as psych Info (100), Google scholar (26,600) and Mendeley (53) showed selfie related research to date. These facts and figures reflect its growing popularity which itself provide enough ground to explore its understanding among its users and explore explanation behind its strong preference over other pictures. Past studies, which attempted to track down the characteristics of users and their motivation behind high selfie posting on social media had borrowed Oxford dictionary definition (Weiser, 2015; Sorokowski et al., 2016; Ma et al., 2017).

These studies had borrowed the general definition of the word selfie more or less conceptualized selfie just as a picture. Whereas some researchers dig deep in understanding selfie phenomenon and realize the significance of the unique relationship which selfie shared with its user (e.g., Cruz and Thornham, 2015; Senft and Baym, 2015; Lim, 2016). Senft and Baym (2015) had attempted to define it more in terms of its usage and process rather than just a taking and posting activity. According to them, “*a selfie is a photographic object that initiates the transmission of human feeling in the form of a relationship (between photographer and photographed, between image and filtering software, between viewer and viewed, between individuals circulating images, between users and social software architectures, etc.).”* (p. 1589). This definition attempted to go beyond just the functional activity of taking a selfie with posting intention but rather focused on the emotional involvement between the selfie and its user and impact of the same. In addition to this, Lim (2016, p. 1775) had also attempted to conceptualized selfie as “*a self expressive photograph featuring the photographer as the primary individual in relation to any secondary products in the background that is consciously created, modified and shared with others to varying degrees, conditional on the dynamic interaction between the personal and situational factors present and facilitated by technology.”* Furthermore, Shipley (2015) also extended this relationship by adding the emphasis on the unique relationship shared between selfie and the smartphones. As Shipley (2015), (p. 404) said, “*Selfies in particular make the mobile phone apparatus into both an extension of the body and a technology of abstracting the self.”*

Furthermore, Svelander and Wiberg (2015) in their article “Practice of selfies” explored ways to provide accurate description of selfie practices and argued that selfie should not be just limited in defining as amateur picture or just the source of self-absorption or narcissism. They further emphasized that selfie is not a simple act of picture taking but a relationship between its owner, selfie, social media platform and their Smartphone. This process may involve steps such as exploring others' selfie, taking one's own selfie, carefully selecting, editing, finalizing and then sharing with others on social media platforms along with expected feedbacks. They also argued that “*selfies need to be understood as a social practice, the result of a long and thoughtful process governed by three mechanisms: social calibration, social probing and social feedback. These three mechanisms of interaction, tightly coupled to their enabling technology, form a basis for interaction design”* (p. 35). To extend this view further, Shipley (2015) also emphasized the influence of technology and internet to create selfie culture among common people and these digital photography and social media circulation was required for its existence and development from a picture into a most prominent trend among people. As Shipley (2015), (p. 404) rightly argued that, “*I contend that the selfie, rather than a singular form of technologically driven self-portraiture, is a multimedia genre of autobiography or memoir that makes the image into the protagonist of stories of his or her own composition. Selfieness is an emotional and semiotic field that emerges through the potential ever-presence of the selfie.”*

This indirectly refer that selfie should not be just seen as an ordinary picture but a process that may understood, created, distributed and conveyed by its owner to his/her audiences. Taking this view forward, the present study stressed on including the “**significance of users' perspective”** in its conceptualization to get a composite understanding of selfie phenomenon. Past studies on selfie did not put much emphasis on defining it, but focused on exploring the relationship between the frequency of selfie posting and various determinants and traits related to its user. The next section discussed the various determinants found responsible for selfie popularity on social media.

### Determinants associated with selfie popularity among its users

Previous studies had examined various factors associated with high popularity of selfie among its users on social media. Since, these studies had conceptualized selfie as a self-photograph which is mostly taken for sharing purposes on diverse social media platforms. Therefore, mostly the research questions on these studies were directed toward prediction and exploration of selfie behavior on social media. Those key research questions were: who shares selfies on social media, why users prefer to share selfies on social media, etc. One of the major personality determinants were suggested to be a strong predictor of high selfie posting behavior on SNS was narcissism (Fox and Rooney, 2015; Sorokowski et al., 2015; Weiser, 2015; Halpern et al., 2016; Arpaci, 2018). Another significant factor “self-presentation” was found to be a strong influence behind such popular selfie posting behavior on social media (Fox and Rooney, 2015; Chua and Chang, 2016; Diefenbach and Christoforakos, 2017). As with technology boon, digital self-portrait popularly termed as “selfie” provides enormous opportunity for tailored self presentation by exploring oneself with numerous selfies (Katz and Crocker, 2015). In addition to this, social media platforms also open the doors of autonomy and control for users to own representation through carefully selected and sometimes edited selfies to be uploaded to seek feedback from others instantly (Chua and Chang, 2016). Sometimes selfies were found to be fit for presenting an “ideal self” to others on social media (Ma et al., 2017). Studies instigated by ‘impression management theories (Goffman, 1959; Leary and Kowalski, 1990) have supported the idea that selfies have given their users colossal opportunity to project an affirmative self-image by presenting a positive physical appearance on social media (Pounders et al., 2016). Furthermore, “self-esteem” an evaluative aspect of individual's self-concept (Gecas, 1982) was also served as a motivation behind selfie posting behavior on social media (Pounders et al., 2016; March and McBean, 2018). Apart from these self-enhancement strategies, there are other motivators played an important role in predicting selfie posting behavior on social media. Selfies also provides a mode for maintaining online social interaction as many selfie users share their selfies on social media to communicate with their peers and audiences about their feelings and whereabouts (Lee et al., 2015). Furthermore, selfies also serves as a good way to preserve their memories on social media in the forms of autobiographies and archiving (Holiday et al., 2016; Sung et al., 2016). It is also proclaimed that a picture is capable of communicating more than words, therefore, selfie might help users to seek attention from their audience and also entertain them (Sung et al., 2016; Kearney, 2018).

On one hand researchers were busy in exploring and examining the selfie posting related behavior, whereas on the other side, few studies had started recognizing the selfie taking aspect of the selfie posting behavior. Researchers had started incorporating selfie taking intentions along with posting intentions on social media. However, the questions which were asked to the participants had framed selfie taking and posting together as a combined activity (Holiday et al., 2016; Shah and Tewari, 2016; Yue et al., 2017). This paper argues that selfie taking activity (the present study termed as “offline” selfie activity) needs to be studied separately from posting activity. The present study gives emphasis to extend the selfie process by drawing attention toward the selfie taking aspect which is the first and fundamental step to selfie posting on social media. Previous studies had explored determinants and motivation behind selfie popularity on social media. These studies took selfie posting frequency as an indicator to demonstrate high selfie engagement of their users on various social media platforms and linked this popular behavior with various psychological concepts and personality factors. The paper would like to focus on keeping a similar account (counting the frequency of selfie taking) for the selfie taking activity of its users. For example, Goffman (1959) self-presentation strategy suggests that in order to present their best side of the self-individual involves in two processes: “Backstage” and “frontstage” performances. Selfie taking could be considered as backstage performance whereas selfie posting could be perceived as frontstage performance in order to maintain social reciprocity with the audience on social media platforms. This “backstage” performance could be engaging, time consuming and high on selfie taking frequency. As to present a perfect “frontstage” performance one needs several rehearsals, similarly to get the perfect selfie to be shared on social media one needs to take several attempts to take good selfies. As Hess (2015) had also discussed about selfie users preference to keep on taking their selfies until they got a perfect angle which ensured a best selfie. Likewise, Palermo (2014) explored two criteria (a flattering facial pose and state of the art technology) for a good selfie which could yield positive likes and comments by the audience on social media. Studies on selfie posting behavior sometimes also mentioned the high selfie taking frequency as compared to posting frequency on social media; however, they did not pay much attention to this discrepancy in selfie emergence process. The larger picture of selfie process may involve three basic steps: (1) selfie taking (2) editing or selecting and (3) posting it on social media. This paper argues that future researches needs to pay attention on selfie taking frequency and its correlates and also conceptualize selfie taking as an activity which might have other motivations except finding a best selfie to share on social media.

This review on determining factors behind selfie popularity among its users on social media suggests an important point that majority of previous studies have only focused on selfie posting frequency and its association with personality factors and psychological concepts. However, the present study argues that the posting frequency on social media could be just a tip of an iceberg and to understand complete selfie phenomenon, both (taking and posting) aspects of selfie need to be acknowledge with their unique significance among selfie users life and should be studied further.

## Objectives of the current research

The present research had identified some gaps and two objectives were examined to address those gaps. By exploring these objectives, the present study makes an attempt to find answers for many questions related to selfie phenomenon and its growing popularity.

- The **first objective** of the study was to explore the selfie phenomenon by adding its users' perspective.
- The **second objective** of this study was exploring the determining factors behind both taking and posting selfies.

## Method

### Participants

A total of 60 college going students took part in this study. These participants were living in Delhi, India. The total sample included 16 (26.67%) male and 44 (73.33%) female respondents. The age range of these participants was 18–29 years with the mean age of 21.62 years and standard deviation 2.07.

### Data collection

The selection of the participants was entirely based on targeting youth (both males and females) with one criterion that they all had Smartphone with front camera and was selfies' users. The recruitment of the participants was done by announcing the requirement of selfie users during various college extracurricular events by the researchers as it could maximize the chances to reach out diverse group of youth at one time. Those students who showed interest in the study were briefed about the purpose of the study. After fixing appointments with those participants, researchers had called each of them for personal interview. All the participants were again briefed about the broad area of the research and were also informed that no financial compensation would be provided to them in lieu of their participation in the study. Informed consent was taken from all the participants before conducting the interviews. The interviews were conducted only after building rapport between the interviewer and interviewee. Comfort and free will to participate in the study was the priority of the researchers during the interview with each participant. Participants were also ensured that their personal information e.g., name, affiliation etc. would not be mentioned in the study and they could anytime withdraw their participation from this study.

The study used semi-structured interview technique to fulfill the objectives of the present research. The semi-structured interview technique was adapted to get maximum information with flexibility and control over the flow of discussion between the interviewee and interviewer (Corbin and Morse, 2003). Therefore, despite having a pre-structure to focus on the key areas of selfie related experiences of the participants. These interviews have given maximum flexibility to the participants to express their experiences in details related to the key questions. They were also allowed and encouraged by the interviewers to mention experiences which they found significantly related to their selfie experiences. All interviews were conducted by third and fourth authors. Before, beginning the final data collection, several preparatory sessions were executed to develop the depth, clarity and experience to conduct unbiased interviews. After designing the broad structure of the interview, two face-to-face pilot interviews were conducted by each of the researcher. After reviewing those pilot interviews, the modifications were further made in agreement with all the authors. With latest modification two-two interviews were again conducted by the researchers. These interviews were again analyzed by all the authors and after ensuring the unbiasedness and covering all the aspects of the study, the final data collection was executed. However, these pilot interviews were not included in the final data analysis. The interviews were average of 25–40 min. The key questions that were asked during interview were: (a) **“about their understanding of selfie”** as previous studies on selfie had paid huge attention on the functionality aspect of selfie and did not much explored the selfie user's perspective toward selfie (e.g., what do you understand about selfie?, how do you define selfie?), (b) their **“average selfie taking ratio”** (e.g., how many selfie you click on daily/weekly/monthly bases?) as reviewing available data based on selfie suggested possible differences in taking and posting frequencies of selfie, therefore this study added this aspect of selfie usage, (c) “**posting frequencies**” of selfie pictures as posting frequency was always found to be linked with various aspect of its users by previous studies; therefore, it was found to be important to keep a record of selfie taking and posting frequency of the same participants, (d) **“the reasons behind both taking and posting selfies”** (e.g., why do you choose to click selfies?; why do you post selfies?). Thus, the selection of these interview questions was based on the literature review and intended to address the gaps and objectives of the study. The complete details of the participants and also their selfie taking and posting frequency are given in the Appendix Table 1.

### Data analysis

All the interviews were first transcribed and then analyzed using the thematic analysis method introduced by Braun and Clarke (2006). As suggested by Braun and Clarke (2006), this method promises flexibility in data analysis with wider applications. The flexible yet clear guidelines of this method allows researcher to explore their own epistemological stand without necessity of keeping any pre-existing theoretical framework. This method can be used to analyze using inductive or deductive approaches. Therefore, it was found to be fit for the present study data analysis as the aim of this study was to explore emerging patterns/themes within the data but not necessary building any theory which require more rigorous data collection and analysis process such as grounded theory etc. This method includes six steps to perform thematic analysis which start with transcription, initial codes generation, collating codes in themes, reviewing and finalizing themes with clear definitions.

## Results

The results based on thematic analysis were discussed in two sections. These two sections were:

1. Conceptualization of selfie phenomenon
2. Determinants of offline (taking) and online (posting) selfie:

### Section 1: conceptualization of selfie phenomenon

This section included themes related to the participants' understanding of selfie. Two major themes (capturing the self and being in control) were found to be significant in representing participants' insight about selfie phenomenon.

#### (1a) capturing the self

This theme is a common understanding regarding selfies among participants. Participants understand selfie as an act of taking picture of oneself with the front camera of Smartphone. However, these selfies are perceived more in terms of “practice of freedom” rather than just a picture taken from front camera.

When **participant 28** was asked about her understanding of selfie, she responded as:

“*…selfie can be referred to as the act of clicking one's own picture especially when no one is around to click it.”*

Similarly, **participant 29** had also perceived selfie as an act of clicking oneself.

“*…Selfie is clicking ourselves to look whichever way we want to with front camera of our mobile.”*

Further, **participant 30** had given an elaborative description of clicking a selfie.

“*…Clicking selfies is very similar to clicking pictures by a cell phone or a camera but the only difference is when we call it selfie it is clicked by the same person who is present in the picture and not by someone else. Nowadays, there are two kinds of camera in cell phones, front and rear camera and we use front camera for clicking selfies…”*

#### (1b) being in control

This theme represents participants' unique and personalized understanding about selfie. According to this theme “being in control” was meant about the control over the creation of self by this distinctive selfie taking process. They can take pictures as per their requirement as with the evolution of selfie feature in Smartphone, one can take multiple pictures without taking other's help. It can offer them a scope for exploring oneself and evolving multiple identities in many creative ways.

**Participant 17** had added technology advancement and freedom of expression in selfie phenomenon:

“*…Whenever selfie is mentioned the first thing that strikes my mind is freedom, don't know why but the feature offered to us by technology is a boon. I used to hear from my grandparents and they tell that whenever they would wish to capture any such precious and priceless moments. they would never ever have got such thing. they say if this was there, they would have also taken truckloads of the pictures as we do. Uhm well for this I would say that we have been made technologically sufficient that we don't need anyone else's help we have freedom which we can avail anytime. whenever we wish that this moment needs to be captured….”*

On the other hand, **participant 27** had talked about the freedom to experiment and explore oneself as the defining feature of selfie for him:

“*Aha….selfies….okay…so…I…ummm.think that it is an empowering tool.I'm not being philosophical but just the way I feel so that grants us control as in we can pick up our phone anytime and click a picture so that we can have the freedom to create like ‘n’ number of identities like an excited face, relaxed one, attitude based expression, looking left and right and it is endless. I sometimes feel I'm technologically backward…umm.because people are damn creative in clicking their selfies…”*

### Section 2a: determinants behind offline (taking) selfie

Five themes (Looks good, keeping memories, mood driven, mirroiring the self and posting on social media) were extracted from the data analysis. These themes were extracted on the bases of its relevance in contrubiting to the determinants behind selfie taking activity by the participants. This qualitative thematic data analysis method gives emphasizes on the relevance, novelty and significance of the content in generating themes. The given five themes were discussed separately below.

#### (I) A selfie is clicked when one “looks good”

Good appearance in a selfie serves as an inspiration for participants to take number of selfies. This theme also reflects the inconspicuous role of selfies in realizing and verifying about the participants exceptional appearances on various occasions. This selfie feature could be seen as in instrument of self-discovery.

As **participant 14** emphasized on her good looks and compliments as strong motivation behind taking selfies.

“*.When I think I look good, I usually click at least one selfie right there. When my friends compliment me that I look good, then it motivates me to take a selfie of me and I take a selfie with my friends too…”*

**Participant 23** also added more to this. As she mentioned:

“*…When I start clicking selfies, and I see in that very moment that I am looking good and selfies are coming out nice, then I feel motivated to click even more selfies…”*

#### (II) Taking a selfie useful for “keeping memories”

Another important factor behind participants' preference to take selfies is the intention to capture moments and save memories forever. Various precious moments and occasions come in one's life and selfies has given opportunity to these participants to preserve them.

**As participant 21** had discussed various occasions where taking selfies were needed for capturing that moment for eternity:

“*…it is like when we have holidays, we have to pass time, like we are going to a party, we feel like taking a selfie, basically that moment gets captured, I think, like when we are in a great moment and there is no one to click your picture, just click a selfie then, and that captures your moment…….for example it's my birthday, then only to capture that moment I click a selfie, any good moment which we want to capture in camera, we take a selfie, nothing more…”*

Furthermore, **participant 34** had also mentioned that selfies helped to capture moments which could be cherished later:

“*… It is actually something we all love, all my friends as well, like my undergraduate friends, we all love clicking selfies, capturing memories. So we click that memories and the view as well. So yeah, that's why we click selfies. It's more of like you know; capturing the moment, that we are together, our happiness, is the thing…”*

#### (III) Taking a selfie is driven by “mood”

This theme had mentioned that taking selfies were also dependent on participants' mood. For example, participants mentioned that when they felt happiness, excitement and cheerfulness, they preferred to take selfies. On the other hand, participants also mentioned that the selfie taking was also helpful in uplifting their mood and boosting their low energy level. In that case, selfie taking activity was found to be strongly influenced by their mood.

**Participant 52** had discussed about her uncontrollable excitement responsible for taking numerous selfies:

“*Ummm…Well…For me, it depends entirely upon my mood per se….when I am in a good mood, then I make everyone around me uncomfortable with clicking selfies… they think I have gone mad! …because it becomes absolutely uncontrollable thing.and I click photos of myself like a maniac….to capture every little expression no matter if I am looking good or…I simply go on and on…Even at that point of time….I do not realistically keep a track on the numbers of selfies being clicked….it's like being in a euphoria…as if you are high on something.…while clicking as I mentioned I really do not keep a track on the number of photos I'm clicking….but yes when it comes to posting ….then…I'm very choosy and select only exclusive pictures by comparing my previous posted pictures on the facebook… Well….first and foremost, according to me.a good mood is very important….if you feel good from within then you will go on and on and on….clicking selfies like crazy….anything….”*

On the other hand, **participant 46** had mentioned that taking selfie can reinforce the mood and energy level:

“*Ah…ummmm…well.like when you are low…then clicking a selfie in my case is very effective as my dejected mood instantly turns into the cheerful one….”*

#### (IV) Mirroring the self

This theme had discussed the usefulness of selfie as mirror for the participants. They take selfies to see themselves or verify their looks and appearance as they do it with the help of a “mirror”.

**Participant 24** mentioned the selfie can act as a mirror and seeing oneself in a mirror (selfie) influenced her to take selfies.

“*Selfie is more like a documentation of life, just like the urge to look into a mirror and sometimes to capture what you see is what motivates me…”*

Furthermore, **participant 28** also emphasized the usefulness of selfie taking availability.

“*.Selfie acts as a portable mirror. For me, appearance matters so it is necessary to look good sometimes. For that selfie helps a lot since mirror isnt always available.”*

#### (V) Posting on social media

This theme reveals participants' intention behind taking selfies for sharing them on various social media platforms like Facebook, Instagram and also for WhatsApp DP.

**Participant 16** mentioned that:

“*My first motivation are my friends, Whenever we are having fun, I usually click selfies and my individual motivation is clicking selfies to make my ‘DP.’ (Display Picture) The environment also sometimes is appropriate for clicking slefies, Crazy times with friends, Also Whenver I see others taking a selfie and enjoying, that motivates me to take a selfie myself…”*

To add more to it, **participant 26** also emphasized the relationship between selfie taking and posting activity.

“*.Posting itself is my motivation, I feel motivated to click selfies as I want to post them on what'sapp, to inform my friends about any event happening in my life, my latest looks and fashion.(posting on social media)…”*

These themes provided a glimpse of various factors behind taking numerous selfies by the participants. These themes also showed that taking selfie had almost replaced the ordinary picture taking act with the uniqueness of selfie. They preferred to take selfies for capturing memories of great moments or capturing their good appearance/looks and sometimes selfies were the outcome of good or bad mood. It can also be utilized as mirror. With such multiple features, selfies had earned a special place in the lives of these participants.

### Section 2b: determinants behind online (posting) selfie

Three themes (Social approval, being the best among the rest and virtual albums to maintain online presence) were found as important factors behind participants' online selfie posting activity. These themes were discussed at length in this section.

#### (I) Social approval

This theme had talked about the strong desire to get positive responses from significant others such as friends, family, and colleagues etc. of these participants. The “social approval” theme also reflected on the participants' careful selection process of selfies to upload on social media which could yield positive feedback and affirmation about them.

**Participant 13** discussed the importance of others' having a good impression of her and a need for positive affirmation as she said:

“*….The subsequent feedback (on posting selfie)….that how are people seeing us… obviously those people might be the best for me….but social approval in a way also counts and not to deny it does matter to me as a person. I guess…in one way it helps us to be connected with our friends…and secondly…that how are we looking from the eyes of others? Whatever picture I would post on a social media platform would yield good amount of likes and comments….As I said…we get a fair idea on how are we looking from the eyes of others….we are being accepted… in terms of good amount of likes…ummmm.ah…well…like it is most of the time the uploading takes place in a shorter time…but your mind wanders around it for like a week.(laughs off).to see what people has commented, reacted…liked etc….”*

In addition to this, **participant 40** had also talked about the need for positive feedback from others:

“*…I feel that to post a picture (selfie) is an expression to call out for feedback. The intent to share is in my opinion motivated by the fact that almost every human being needs social reinforcement. I feel that in the digital world pictures are a richer type of content than text is since pictures do not only convey a message but also a glimpse on the context like the mood etc. etc. 'A picture says more than a 1000 words' and I think the combination of the urge to get positive affirmation, and the personal intent to become very good (a master) at something can be called the urge for approval….also a picture has a greater potential to convey a subtle message and call for more complex feedback…. I believe people do not only post comment to receive spoken, concrete feedback. …….I think people are also motivated to leave an impression about them. On forums like Facebook, posting selfie to me is a subtle form of social approval. I think, I moreover, post a photo because I want positive affirmation about how I live…”*

#### (II) Being the best among the rest

This theme reflect upon the strong urge of the participants' to present themselves different and extraordinary by posting their carefully selected best selfies on SNS.

As p**articipant 1** mentioned that

“*…Most importantly, looking beautiful in selfie and it should be best from the rest. Like I just select the best photos and then post it. I do not just randomly upload, like ok… I'm looking ok in this picture so let's post it. NO! I'm like, I click at least 15–20 photos and like when I am taking a selfie and then I am also comparing it that which photo is good. Then I post it! And if it's not good then I do not post it… also peer-pressure is there that okay they are posting the picture then let me too post the picture. Because there is something like if someone is doing that thing then you will also do that thing…”*

Further, **participant 50** also shared her good looking selfie as a motivation to post:

“*…I think I post selfies when I'm looking and feeling my best. I'm usually most confident on posting selfies like after I have a new hairdo for example or when I have fabulous makeup or outfit. It's kind of like a "hey look how hot I look with my new hairstyle….then it makes me feel fabulous….”*

#### (III) To maintain online presence

This theme represented the willingness of participants to post their selfies in order to add information about their whereabouts in their social media accounts which could be accessed by others at any point of time. Participants had also mentioned that online selfie posting activity help them in keeping their social media account lively so that people would not forget their existance. It was also found to be helpful way to communicate and connect with others.

**Participant 51** discussed the significance of posting online selfies at length:

“*. I'm very choosy about my selfies because each could not carry potential for Facebook profile picture… I like to keep on changing so that my personal page does not go static…… Not everyone can afford a celebrity photographer like daboo ratnani…that's why I ensure to take best picture at my level… each carefully chosen image suppose to reflect a mood and to communicate something about myself……It is self-involved but it is my own page and my own self that I'm trying to express and interpret…”*

Furthermore, **participant 13** also added the relevance of keeping virtual albums:

“*.“….I think that the way in which people click and upload their selfies….doing and enjoying their life in their own way. Somewhere it would also ring the bell at the back of our mind that, Are we getting isolated? Somewhere are people forgetting about us and this is why clicking a selfie and posting it becomes a priority…”*

She further added:

“*…In this fast paced world…it is not at all possible to be in touch with almost everyone….so it's a good way to keep yourself and others updated on how you have been for such a long time and how are you doing in life……”*

These three major themes revealed an important and meticulous process of choosing best selfie. They posted it either to validate their good looks and appearances from positive feedbacks and comments on social media. They might be simply intended to get attention or keep their presence and also seek social approval of others.

## Discussion

The present study is a preliminary attempt to explore the contributing factors in both offline (taking) and online (posting on SNS) selfie involvement among youth population. This offline selfie involvement could be another area which may provide holistic view of selfie process ranging from taking selfies to selfie posting on social media. Furthermore, the present study had also attempted to focus on extending the selfie phenomenon by incorporating the perspective of its users. The findings of this study argue that selfies' multifaceted nature which made it unique from other pictures and hence added to its vast popularity. These three aspects (meaning of selfie, taking of selfies and posting on social media) of selfie process could help to unravel the reasoning behind its vast popularity among its users.

### What is selfie

This selfie phenomenon had gained huge academic interest in the last few years. However, it was mostly understood from the general definition of the word “selfie” given by Oxford dictionary in 2013. The present study emphasized on extending this basic definition further as it does not provide enough account for the meaning, purpose and uses it serve for selfie users (Warfield, 2014). The present study also argued that selfie should be seen distinctive in nature as it is more than a picture or rather it could be called a “story” created by the user to connect and communicate with his/her audience (Shipley, 2015). Results of the present study supported this argument and showed that participants understood selfie in two important ways: **First:** “selfie” as a medium to capture oneself with the help of front camera of the Smartphone which is handy, accessible and perceived as mode for recreating the self (Belk, 2014). One important aspect of selfie understanding about the participants was noticed that they do perceive selfie as an act of taking one's picture by oneself, but its sharing on social media was not immediately linked with selfie understanding.

**Second:** Participants had also perceived “selfie” as not just a picture but a mode to give them immense opportunity to be independent or in control. Thus, selfies become a medium which allows the user to be self-reliant, be at ease while capturing the moment, be comfortable in posing and also have the space for experimentation while taking pictures. These selfies should be seen as a versatile and powerful tool for self-discovery, creating desired identity and having control over the images that one wants to create (Belk, 2013; Kwon and Kwon, 2014). This idea could be supported by (Senft and Baym, 2015), p. 1589) who rightly signifies “*selfie as a human agency*” as it is not captured but created (carefully constructed and selected) by its user which also communicate his/her feeling and emotions.

### Why they take selfies

This multifaceted nature of selfie may have influenced the participants to prefer selfies and could be seen as a reason behind its popularity. Thematic analysis suggested five important factors behind the offline selfie involvement of participants. ***First: “looks good”*** factor suggested that these participants were motivated to capture their looks by taking numerous selfies on various occasions when they found themselves in good looks. This finding is also supported by the study done by Dutta et al. (2016) on Indian school going adolescents. As they found that 70 percent of the total adolescents participated in the study were motivated to take selfies inspired by their good looks and dressing done for special occasions. The selfie taking activity influenced by physical appearance could be seen as a gateway to strive for “ideal or desired self”. This ideal or desired self could be derived by body politics, desired identity construction or maintaining beauty and physical appearances to reach the global standards (Swaminathan, 2014; Tiidenberg and Cruz, 2015).

***Second: “keeping memories”*** factor showed that participants wanted to keep memories of special occasions and moments of oneself and also with significant others (Holiday et al., 2016). To preserve the significant moments alone and also with others with comfort motivates the participants to choose selfies for capturing such occasions (Shah and Tewari, 2016). *T**hird: “mood driven”*** factor had influenced participants' preferences on taking selfies in big amount. They either interested to capture those natural expressions on the face or to elate the low mood and boost their energy level. This factor provides a positive outlook in linking selfie with positive outcomes such enhancing emotional wellbeing of selfie users (Chen et al., 2016). Furthermore, ***fourth:* “mirroring the self**” showed a different utilization of selfie (Warfield, 2014). The selfies are being utilized as mirror for verifying one's looks and appearance as one does through using a mirror (Shah and Tewari, 2016). Lastly, **fifth: “intention *for posting on SNS”*** was found as a significant motivating factor behind selfie offline (taking) engagement among participants. This driving force behind selfie taking involvement does not require further explanation as all the social media platforms with increasing numbers of selfie sharing speaks for itself. To add more to this, during interview sessions many of the participants had mentioned that it takes time to click, select selfies particularity for sharing purposes. Only after careful scrutiny and inspection; they chose the best selfie to post on social media. Warfield (2016) also mentioned the tedious process to get “good selfie” which include arranging important amenities such as best dress, perfect light, best pose, and hair makeup, further series of takes to get the “good selfie” to post on SNS.

Along with recognizing the significance of selfie posting intention, the present study argues that selfie taking activity is not limited to selfie posting behavior. There are other motivations and determining factors works behind selfie taking involvement except selfie posting intention. The Appendix Table 1 reported *participants' **selfie taking and posting ratio***. Differences could be seen in participants' selfie taking and posting frequency, which suggests that selfie taking activity serves a multipurpose medium for them. The differences in selfie taking and selfie posting ratio mentioned in few previous studies were largely went unnoticed (Holiday et al., 2016; Shah and Tewari, 2016; Yue et al., 2017). This understated representation of selfie taking and posting differences in those studies indirectly suggests that these differences are largely insignificant or it may be a result of the journey to achieve best selfie to present oneself in a desired manner to their audiences.

### Why they share selfies on social media

Further, results of the present study also showed that participants [who like to capture their creative, emotion loaded selfie along with representing important moments and occasions] would also like to share some of their selfies on social media. The present study found three determinants that influenced the participants to share their selfie on social media. ***First: “social approval”*:** This theme reflects participants' urge for social validation in the forms of likes and comments on their selfie posts. Participants mentioned that they wanted be assured about their views on life events, looks and their abilities which they might be exhibiting through their selfie post. The assurance and validation through positive comments and likes might help in strengthening their self-esteem (Gonzales and Hancock, 2011; Pounders et al., 2016). ***Second: “being the best among the rest”*** factor showed that selfies can be used to fulfill one's desire to be unique and extraordinary. To attain this purpose only best selected selfies were posted to yield positive feedback from others. The significance of positive feedbacks was supported by the impression management theories which give emphasis on the use various strategies to present the best side of their self to others (Goffman, 1959; Markus and Nurius, 1986). Furthermore, studies on self-presentation had also shown the significance of others feedback and positive affirmation on one's post on SNS (Chua and Chang, 2016). Furthermore, ***third: “to maintain online presence”*** factor could readdress selfies from seeing as picture to become communication tool (Sung et al., 2016; Kearney, 2018) and also made selfie as a useful tool to serve various purposes such as maintaining their presence among the other users. Posting selfies on their social media accounts could help to keep their account lively for viewers.

## Limitations and future directions

The present study had chosen a qualitative design that allows in unearthing of vital and valuable aspects of selfie conceptualization and its popularity among youths. The qualitative design in general, aims to get the nuanced details of any construct; therefore, the range of sample is not predetermined (Braun and Clarke, 2013). In respect to the quantitative research designs, this study carries a small sample size which will lack the vital aim of generalization, however provide promising ground for future exploration. Another limitation of this study was imbalanced male and female participants. Since, the researchers had recruited those who were willing to participate (in our case, we were approached by more females participants) and gave consent to participate. The data collection process could not extend until it had proportionally equal males and females due to time and money constrains. Future studies with representative and gender balanced sample would help to address these limitations and also extend the application of these findings further.

Despite certain limitations, this study is a preliminary attempt to explore certain aspect of selfie concept and its increasing popularity which had not been much explored. The findings of the study invite attention of researchers working on selfie posting behavior to explore an important aspect of selfie taking. However, still some areas are warrant and needed to be explored further to develop deeper understanding of selfie popularity. For example, apart from selfie posting aspect, selfie taking and the selection of selfies to share on social media is also an important for understanding the selfie process. A survey done by The Renfrew Center Foundation (2014) reported that around 85 percentage of youth (18 years and above) post their pictures (selfies) online and around 50 percent of these youth edit their pictures to make them look better than actual. Chae (2017) had explored the selfie editing behavior and found that selfie editing is motivated by the desire to be ideal for online self-presentation. In the light of these evidences, it seems important for future researches to examine all parts (taking, selecting, editing and online selfie posting) of selfie process. This study had also attempted to extend selfie conceptualization by adding the selfie user's perspective. The future researchers should expand further this dynamic relationship between selfie and its users' on various grounds such as gender, age, role of peers, and cultural setting.

## Conclusion

The findings of this preliminarily study to understand selfie process suggested that selfie need to be studied from both offline and online perspectives. The present study found several factors which played an important role in selfie offline and online involvement which may or may not be a sign of negative personality traits or as a filler that satisfies the needs of individuals [whether it is seeking peers' approval, making social comparisons, constantly making validation cues for one's beauty and looks or seen as tool for enhancing low self-esteem] as the findings of the past studies suggested behind the user's online selfie involvement. This study suggests a parallel path to explore selfie popularity by incorporating users understanding of selfie. Perhaps, selfies have become the medium between the user and user's self to connect themselves in a healthy way as they get a chance to see them, explore them and be with them, perhaps with rear camera that opportunity was missing.

## Ethics statement

An ethics committee approval was not requested. This study did not include any human experimentation or deception about the study purpose. Participants were prior informed about the research purpose and after their consent and approval, they participated in the interview. Participants were free to stop participation at any time.

## Author contributions

The whole study was designed by SaS and PU. The interviews were conducted and transcribed by ShS and KG. The data analysis was done by PU, however feedback from the remaining authors was also incorporated in various stages of data analysis. The first draft of the manuscript was prepared by SaS and PU. After several revisions based on inputs from all authors, a final draft of the manuscript was prepared by SaS and PU and approval was taken from other authors for submission.

### Conflict of interest statement

The authors declare that the research was conducted in the absence of any commercial or financial relationships that could be construed as a potential conflict of interest.
