# Supplementary material for: Exploring Factors Behind Offline and Online Selfie Popularity Among Youth in India
Source: Front Psychol. 2018 Aug 7;9:1403. doi: 10.3389/fpsyg.2018.01403 (PMC6090501; doi:10.3389/fpsyg.2018.01403)
Supplement: Supplementary file 1 [file Table_1.docx]

**Appendix table 1**

| **Participant ID** | **Gender** | **Age** | **Educational Qualification** | **Place** | **Selfie (taking)** | **Selfie (posting)** |
| --- | --- | --- | --- | --- | --- | --- |
| P1 | Female | 18 | Undergraduate | Delhi | 3 in a month | 1in 4-5month |
| P2 | Female | 19 | Undergraduate | Delhi | 30-40 in a day | 8 in a day |
| P3 | Male | 23 | Postgraduate | Delhi | occasionally | Mostly |
| P4 | Female | 19 | Undergraduate | Delhi | occasionally | Rarely |
| P5 | Female | 22 | Postgraduate | Delhi | 1in 2-days | Rarely |
| P6 | Female | 22 | Post Graduate | Delhi | 4 times^*^ in a week | 1 times^*^ in a week |
| P7 | Female | 24 | Postgraduate | Delhi | occasionally | occasionally |
| P8 | Male | 21 | Postgraduate | Delhi | NA^**^ | NA^**^ |
| P9 | Female | 22 | Undergraduate | Delhi | occasionally | 1-2 times^*^ in 5-months |
| P10 | Female | 21 | Undergraduate | Delhi | 2 in a day | 10 in a month |
| P11 | Female | 22 | Undergraduate | Delhi | NA^**^ (monthly basis) | NA^**^ |
| P12 | Female | 18 | Undergraduate | Delhi | 3 in a day | 1 in a day |
| P13 | Female | 24 | Postgraduate | Delhi | 5-6 times^*^ in a day | NA^**^ |
| P14 | Male | 19 | Undergraduate | Delhi | 10 in a day | 12 in a month |
| P15 | Female | 23 | Undergraduate | Delhi | NA^**^ (each day) | NA^**^ |
| P16 | Male | 23 | Postgraduate | Delhi | 500-600 in a month | NA^**^ |
| P17 | Female | 21 | Postgraduate | Delhi | 7 times^*^ in a month | Occasionally |
| P18 | Female | 19 | Undergraduate | Delhi | 6 in an hour | Rarely |
| P19 | Female | 21 | Undergraduate | Delhi | 4 in a day | NA^**^ |
| P20 | Female | 23 | Postgraduate | Delhi | 10-20 in a month | Rarely |
| P21 | Female | 24 | Post Graduate | Delhi | 2 in a day | no one |
| P22 | Female | 20 | Undergraduate | Delhi | 30-35 at a time | 1-2 at a time |
| P23 | Female | 19 | Undergraduate | Delhi | 10 in a day | Rarely |
| P24 | Female | 22 | Postgraduate | Delhi | 15 in a day | 2 in a day |
| P25 | Female | 21 | Postgraduate | Delhi | whole day | 4 in a week |
| P26 | Female | 22 | Undergraduate | Delhi | 40-50 in a month | 15 in a month |
| P27 | Male | 18 | Undergraduate | Delhi | 12-15 in a week | NA^**^ |
| P28 | Female | 22 | Post Graduate | Delhi | 500 in a month | 7 in a month |
| P29 | Female | 19 | Undergraduate | Delhi | 3-4 times^*^ in a month | NA^**^ |
| P30 | Female | 22 | Post Graduate | Delhi | 7-8 in a month | 1 in a month |
| P31 | Female | 23 | Postgraduate | Delhi | Occasionally(3-4 times^*^) | NA^**^ |
| P32 | Male | 22 | Post Graduate | Delhi | 20 in a month | very rarely |
| P33 | Female | 21 | Postgraduate | Delhi | 5 in a day | 1in a day |
| P34 | Female | 22 | Postgraduate | Delhi | 4-5 in a day | 1-2 in a day |
| P35 | Female | 29 | Graduate | Delhi | 4 in a week | NA^**^ |
| P36 | Female | 22 | Postgraduate | Delhi | once in month | NA^**^ |
| P37 | Female | 24 | Postgraduate | Delhi | 4 in a day | NA^**^ |
| P38 | Male | 23 | Undergraduate | Delhi | NA^**^ | NA^**^ |
| P39 | Female | 19 | Undergraduate | Delhi | 10-15 in a day | Rarely |
| P40 | Female | 24 | Postgraduate | Delhi | 6-7 times^*^ in a month | NA^**^ |
| P41 | Male | 22 | Undergraduate | Delhi | NA^**^ | NA^**^ |
| P42 | Male | 21 | Undergraduate | Delhi | 12 in a month | NA^**^ |
| P43 | Male | 21 | Undergraduate | Delhi | NA^**^ | NA^**^ |
| P44 | Male | 23 | Undergraduate | Delhi | 15-20 in a daily | NA^**^ |
| P45 | Female | 24 | Postgraduate | Delhi | unlimited at a time | once in month |
| P46 | Female | 19 | Undergraduate | Delhi | 15(monthly) | NA^**^ |
| P47 | Female | 19 | Undergraduate | Delhi | 6-7 in a day | 6-7 in a day |
| P48 | Male | 23 | Postgraduate | Delhi | NA^**^ | NA^**^ |
| P49 | Female | 22 | Postgraduate | Delhi | NA^**^ | NA^**^ |
| P50 | Female | 19 | Undergraduate | Delhi | 4-5 times^*^ in a month | NA^**^ |
| P51 | Male | 21 | Undergraduate | Delhi | 20-30 in a day | NA^**^ |
| P52 | Female | 25 | Undergraduate | Delhi | no track(keep on going) | 2 in a month |
| P53 | Male | 20 | Undergraduate | Delhi | NA^**^ | NA^**^ |
| P54 | Male | 22 | Postgraduate | Delhi | lot of (unlimited) | NA^**^ |
| P55 | Male | 21 | Undergraduate | Delhi | 50-60 in a day | NA^**^ |
| P56 | Female | 20 | Undergraduate | Delhi | 20-30 in a day | NA^**^ |
| P57 | Female | 24 | Postgraduate | Delhi | once in a month | very rarely |
| P58 | Female | 21 | Undergraduate | Delhi | 20 in a day | 6-7 in a day |
| P59 | Female | 24 | Postgraduate | Delhi | 7-8 in a month | NA^**^ |
| P60 | Female | 24 | Postgraduate | Delhi | 20-30 in a day | 3-4 in a day |

times^*^  represents 3-4 selfies in single occasion e.g. 5 times means 5x(3-4 selfies)= 15-20 selfies.

NA^**^ represents the area where participants did not provide exact counting however, they click/post their selfies.
